# Case Report: Co-infection of mucormycosis with mycoplasma pneumoniae in children with diabetes mellitus: report of two rare cases

**DOI:** 10.3389/fped.2025.1516117

**Published:** 2025-05-12

**Authors:** Yipaguli Simijiang, Fawudan Abudu, Yanfei Cui, Abulikemu Abudujielili

**Affiliations:** ^1^ Department of Critical Care Medicine, Pediatric Research Institute of Xinjiang Uygur Autonomous Region, Children's Hospital of Xinjiang Uygur Autonomous Region, Xinjiang Hospital of Beijing Children's Hospital, The Seventh People’s Hospital of Xinjiang Uygur Autonomous Region, Urumqi, Xinjiang, China; ^2^ Department of Cardiothoracic Surgery, Pediatric Research Institute of Xinjiang Uygur Autonomous Region, Children's Hospital of Xinjiang Uygur Autonomous Region, Xinjiang Hospital of Beijing Children's Hospital, The Seventh People’s Hospital of Xinjiang Uygur Autonomous Region, Urumqi, Xinjiang, China

**Keywords:** mucormycosis, mycoplasma pneumoniae, diabetes mellitus, pediatric population, co-infection

## Abstract

**Background:**

Mucormycosis is a rare disease characterized by its highly vascular invasiveness, rapid progression, and high mortality rate. Historically, reports on mucormycosis have been concentrated in the adult population, with few cases documented in pediatrics. Notably, there have been no reports of mucormycosis in either adults or children during the prevalence of mycoplasma pneumoniae.

**Case presentation:**

This paper presents two cases of newly diagnosed diabetic children who developed concurrent mucormycosis during mycoplasma pneumoniae infection. Both patients received aggressive antifungal therapy, with one surviving and the other succumbing to the disease.

**Conclusions:**

The paper discusses the diagnostic and therapeutic challenges of mucormycosis in patients with diabetes and concurrent mycoplasma pneumoniae, emphasizing the need for more proactive identification of infecting pathogens in the diabetic population during mycoplasma pneumoniae outbreaks. When routine anti-mycoplasma treatments are ineffective and chest enhanced CT scans reveal pulmonary vascular destruction, the possibility of mucormycosis should be considered. Particularly in patients with a history of corticosteroid use, clinical suspicion should be heightened. Concurrent mucormycosis infection may worsen patient outcomes, and further clinical exploration of the pathogenesis and treatment recommendations for mucormycosis associated with mycoplasma pneumoniae is warranted.

## Introduction

In 2023, Mycoplasma pneumoniae infections began to surge globally, particularly among children, with widespread outbreaks reported in countries such as France and the Netherlands (1, 2). Denmark had the highest number of detected cases (3). In China, there has been an increase in cases of severe, refractory macrolide-resistant Mycoplasma pneumoniae infections, posing a significant threat to the health of the pediatric population (4). During this outbreak, the most common co-infections included rhinovirus, influenza A virus, respiratory syncytial virus, human coronavirus OC43, influenza B virus, and interstitial pneumonia virus (1). Co-infection with mucormycosis in the same host is rare. Mucormycosis tends to occur in patients with diabetes or with diabetic ketoacidosis (5). Due to the nonspecific clinical symptoms and imaging findings following infection, diagnosis is often delayed, and the prognosis once infected is poor, warranting attention (6). Against the backdrop of mycoplasma pneumonia, we report for the first time two cases of newly diagnosed diabetic children with co-infection of mucormycosis.

## Case presentation

### Case 1

**Figure 1:**
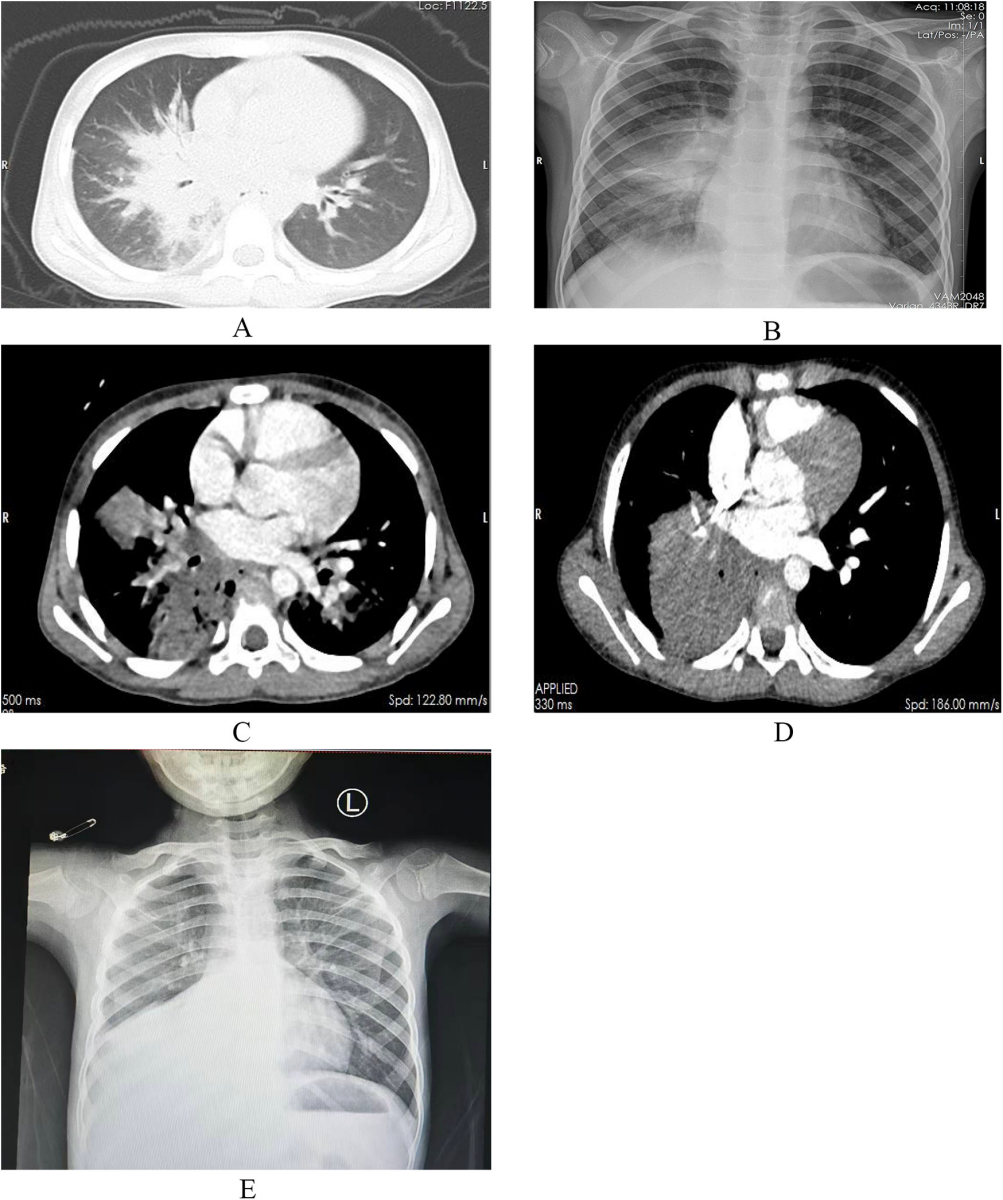
Inflammatory changes in the right middle and lower lobes and the left lower lobe, with associated consolidation in the right lung **(A)**. Right lung pneumonia with partial consolidation **(B)**. Bilateral multiple patchy and large confluent opacities with surrounding halo signs, possible involvement of the pulmonary arteries and veins in the right lower lobe, and local pulmonary tissue with a tendency toward necrosis **(C)**. Compared with the previous study, patchy and flocculent opacities in the right middle lobe and left lower lobe are not visualized, and the consolidation in the outer segment of the right middle lobe and the right lower lobe has significantly increased. The degree of obstruction of the pulmonary arteries and veins remains unchanged **(D)**. Right lung pneumonia with partial consolidation **(E).**

**Figure 2:**
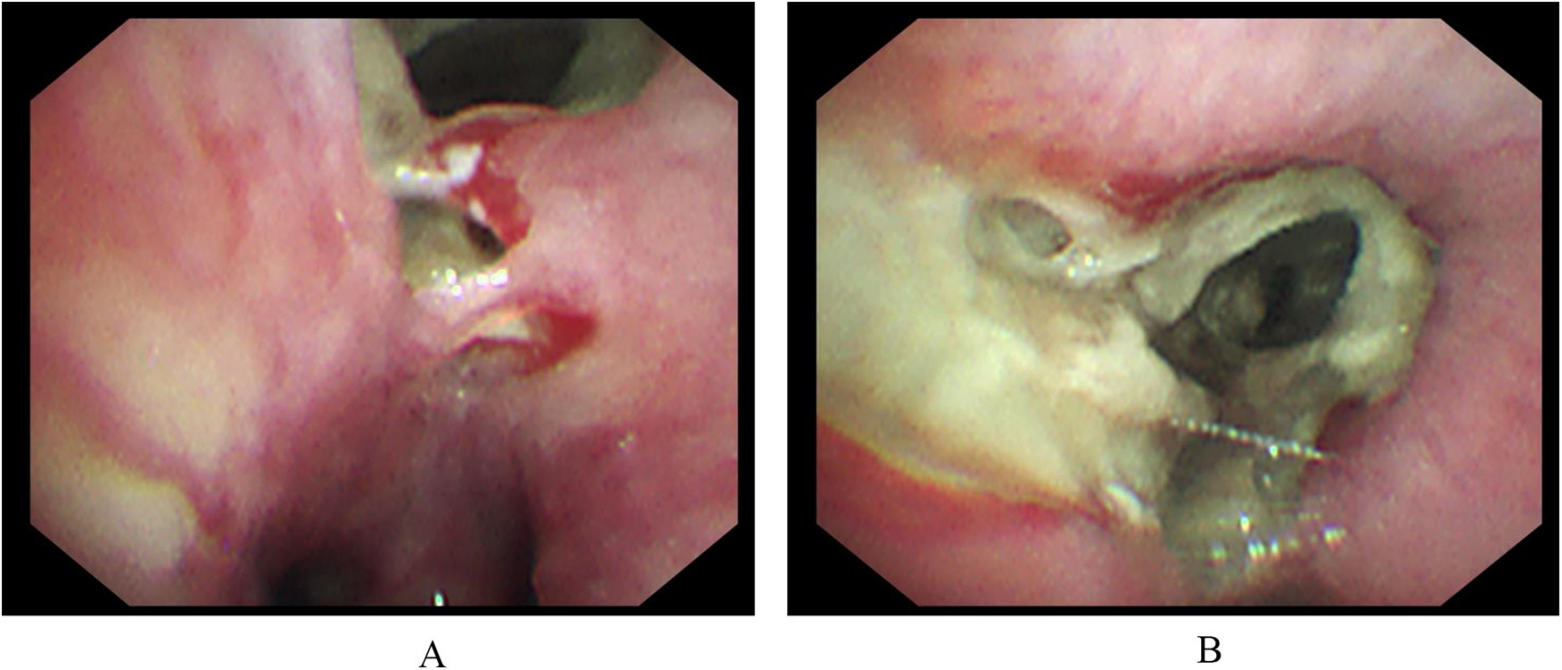
Hemorrhage is visible under electronic bronchoscopy **(A)**. The right intermediate bronchus and various subsegments of the right middle and lower lobes are narrowed by brown mucous plugs, leading to luminal stenosis **(B)**.

On December 20, 2023, a 6-year-old boy presented to our Endocrinology and Metabolism Department with a 1-month history of polydipsia, polyuria, and polyphagia, a 2-week history of cough, and a 6-day history of fever. One week prior, he had been seen for altered mental status, and laboratory tests were consistent with diabetic ketoacidosis. Due to a poor response to treatment, he was referred to our hospital. Upon admission, the child's temperature was 37.8°C, heart rate was 124 beats per minute, respiratory rate was 23 breaths per minute, and blood pressure was 105/65 mmHg. Auscultation of both lungs revealed rales, and the rest of the physical examination was unremarkable. Abnormal laboratory results included a white blood cell count of 14.68 × 10^9^/L, a neutrophil count of 10.12 × 10^9^/L, a C-reactive protein level of 52.26 mg/L, a procalcitonin level of 0.62 ng/ml, an interleukin-6 level of 104.3 pg/ml, and an erythrocyte sedimentation rate of 120 mm/h. PCR testing for respiratory pathogens revealed positivity for influenza A virus and negativity for Mycoplasma pneumoniae nucleic acid, with Epstein–Barr virus IgM antibody also testing positive. A chest CT scan (Figure 1A) showed pulmonary consolidation. Given the clinical presentation and laboratory findings, a diagnosis of diabetes mellitus complicated by pneumonia was made. The child was treated with short-acting and long-acting insulin to control blood glucose, peramivir at a dose of 10 mg/kg per day for influenza virus, and acyclovir at a dose of 30 mg/kg per day in three divided doses for Epstein–Barr virus. Bronchodilators and budesonide suspension were also administered to improve airway conditions. Given the prevailing season for Mycoplasma pneumoniae and influenza virus infections, concurrent Mycoplasma pneumoniae and viral pneumonia were suspected. However, the family declined targeted respiratory pathogen testing. Empirical treatment with azithromycin was initiated at a dose of 10 mg/kg/day to control the condition. After 1 week of treatment, the child's laboratory indicators improved, but he still had fever and a worsening cough. Abnormal coagulation indicators, combined with the progression of radiological findings, led to the diagnosis of severe macrolide-resistant Mycoplasma pneumoniae pneumonia. Heparin sodium was added for anticoagulation at a dose of 30 units/kg per day in three divided doses, and methylprednisolone sodium succinate was administered to reduce the inflammatory response at a dose of 2 mg/kg per day. Due to the worsening condition, the child was transferred to the Intensive Care Unit. A follow-up chest x-ray indicated right lung consolidation (Figure 1B), and bronchoscopy was performed due to suspicion of mucous plug obstruction, which revealed bleeding and thick secretions blocking the airways (Figure 2). Due to tissue fragility and bleeding risk, further biopsies were avoided. Financial constraints precluded detailed analyses. Microscopically, the lesion appeared infectious. Bronchoalveolar lavage fluid was sequenced to identify the pathogen. The sequencing revealed 15,857 Rhizopus sequences, 47 Mycoplasma pneumoniae sequences, 214,603 human parainfluenza virus type 3 sequences, 39,846 influenza B virus sequences, 4 rhinovirus B sequences, 1 influenza A virus sequence, and 3 human herpesvirus 6B sequences. The child had clear evidence of viral infection, and Mycoplasma pneumoniae pneumonia is often associated with viral infections. The patient exhibited host factors and had sufficient microbiological evidence, resulting in a diagnosis of co-infection with Mycoplasma pneumoniae and mucormycosis. After the diagnosis was confirmed, the treatment plan was optimized. Acyclovir was discontinued, and the oseltamivir course was deemed adequate. Surgical intervention was recommended for the mucormycosis, but the family declined, opting instead for medical therapy alone. Treatment included liposomal amphotericin B, but due to a drug shortage after 2 days of use, it was replaced with amphotericin B deoxycholate at a dose of 0.7 mg/kg per day, along with local inhalation at a dose of 10 mg per day in two divided doses. Posaconazole suspension was actively used at a dose of 12 mg/kg per day in three divided doses. Doxycycline at a dose of 4 mg/kg/day, divided into two doses. Insulin dosage was adjusted based on blood glucose levels. After 2 days of these treatment measures, the child's fever subsided, and abnormal indicators improved upon re-examination. After 4 days, a chest enhanced CT showed bilateral pulmonary consolidation and pulmonary vascular involvement (Figure 1C). On day 47 of hospitalization, a follow-up contrast-enhanced chest CT (Figure 1D) revealed no significant improvement in the pulmonary condition compared with the previous imaging. After 57 days of treatment, the child was transferred to a Local hospital for ongoing supportive care. During treatment, organ function was normal except for hypokalemia, which improved with potassium supplementation. After 30 days, a chest x-ray showed no progression in right lung consolidation (Figure 1E). Due to resource limitations, no chest enhanced CT was performed. After 45 days, the child's blood glucose was controlled, with no fever or respiratory symptoms, and all lab indicators were good. The family chose to discharge the child for home observation. They later discontinued posaconazole suspension due to cost and did not pursue further imaging. To date, the child's general condition remains good.

### Case 2

**Figure 3:**
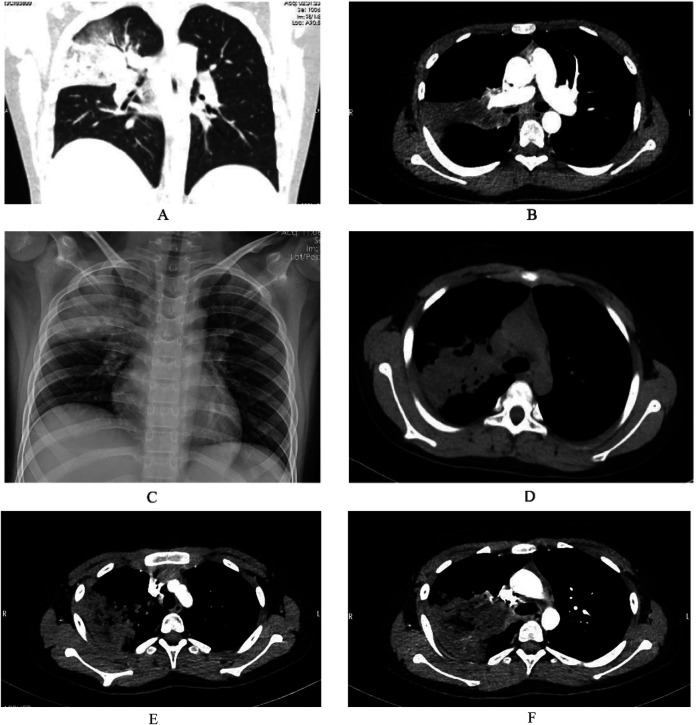
Partial consolidation in the right upper lobe, subpleural nodular lesions in the basal segments of both lower lobes, stenosis at the origin of the right upper lobe bronchus, and multiple enlarged lymph nodes in the mediastinum and right hilum **(A)**. Pneumonia in the right upper lobe with partial consolidation, containing irregular lower-density areas, no significant enhancement on contrast scanning, stenosis at the origin of the bronchus in the right upper lobe, localized air trapping, and reduced branches of the right upper lobe pulmonary artery. There is no contrast filling in the distal right upper lobe pulmonary artery **(B)**. A large confluent consolidation in the upper right lung field with uneven density compared to the previous study **(C)**. Compared with the previous CT, the patchy and consolidated areas in the right upper lobe have decreased in size, with new circular and irregular cavitary formations, slightly increased density, new nodules, and faint patchy shadows in the right lower lobe. Subpleural nodules are noted in the outer basal segments of both lower lobes, and the local narrowing of the right mainstem bronchus remains unchanged as before **(D)**. Reduced branching of the right upper lobe artery, distal filling defects in the posterior segmental pulmonary artery of the right upper lobe **(E)**, and vascular destruction changes **(F).**

On March 14, 2023, an 11-year-old boy presented with a 3-week history of intermittent fever and a 2-week history of cough and hyperglycemia. Two weeks prior, during treatment at a local hospital, hyperglycemia was detected, and laboratory tests were consistent with diabetes mellitus. Additional abnormal laboratory results included a C-reactive protein (CRP) level of 121.49 mg/L and an interleukin-6 (IL-6) level of 41.7 pg/ml. On day 10 of illness, chest CT demonstrated right upper lobe pneumonia, segmental atelectasis, and bilateral pleural effusion. Bronchoscopy revealed caseous necrosis in the right main bronchus, resulting in obstruction of the right upper lobe. Owing to limitations in local treatment facilities, further detailed examinations were not conducted. Aspergillus galactomannan (GM) test was negative, and a diagnosis of severe pneumonia was established, although the causative pathogen was not identified. The patient was treated with piperacillin-tazobactam for infection, methylprednisolone sodium succinate to reduce the inflammatory response, insulin to control blood glucose, and budesonide suspension for inhalation. Initial control of blood glucose was achieved, but respiratory symptoms did not improve, leading to admission to our hospital. On admission, the patient's temperature was 38°C, heart rate was 120 beats per minute, respiratory rate was 24 breaths per minute, and blood pressure was 105/68 mmHg. Auscultation of both lungs revealed moist rales, with no other significant findings on physical examination. Additional abnormal laboratory results included a white blood cell count of 12.98 × 10^9^/L, a neutrophil count of 8.93 × 10^9^/L, a CRP level of 37.14 mg/L, a procalcitonin level of 0.12 ng/ml, an erythrocyte sedimentation rate (ESR) of 62 mm/h, and a positive passive agglutination test for Mycoplasma antibody at a titer of 1:160. Chest CT showed partial consolidation of the right upper lobe (Figure 3A), and Mycoplasma pneumoniae was diagnosed. Clarithromycin (500 mg daily, divided into two doses) was administered with local nebulized medication inhalation and insulin therapy for glycemic control. A subsequent contrast-enhanced chest CT revealed partial consolidation in the right upper lobe, bronchial lumen narrowing, and vascular changes (Figures 3B,E,F). Since tuberculosis smear and TSPOT tests were negative, and the patient had no symptoms of tuberculosis, the diagnosis of tuberculosis was insufficient. Fungal elements were identified in sputum smear specimens, Follow-up chest radiography demonstrated increased heterogeneity in the density of the areas of consolidation compared with prior imaging. Additionally, imaging revealed a trend toward increased local airway obstruction (Figure 3C), which is highly suggestive of mucormycosis. Given the high risk of tissue biopsy for the patient, a multidisciplinary discussion involving the departments of critical care medicine, clinical pharmacy, radiology, thoracic surgery, and anesthesiology was held. The consensus was that the patient could be diagnosed with mucormycosis and recommended aggressive anti-mucormycosis and anti-Mycoplasma treatment, with bronchoscopy potentially causing more harm than benefit. After being informed of the discussion, the family agreed to medical treatment. Due to the unavailability of liposomal amphotericin B and posaconazole suspension, amphotericin B deoxycholate was used, starting at a dose of 0.1 mg/kg/day and gradually increasing to 0.7 mg/kg/day. After 17 days of treatment, chest imaging showed a reduction in the area of consolidation with new circular and irregular cavities (Figure 3D). Despite antifungal and anti-Mycoplasma treatment, on the 18th day of hospitalization, the patient experienced epistaxis, followed by severe coughing, massive hemoptysis, and uncontrolled bleeding from the mouth and nose, leading to death despite resuscitation efforts.

## Discussion and conclusion

In recent years, there has been a noted increase in the global incidence of mucormycosis (7). New high-risk populations have been identified, such as patients with concurrent respiratory viral infections, who exhibit significantly higher rates of fungal coinfections and often suffer severe consequences (8). During the COVID-19 pandemic, an outbreak of COVID-19 associated rhino-orbital-cerebral mucormycosis occurred in the Indian region, with a mortality rate as high as 50% (9). The emergence of this condition is thought to be related to poorly controlled diabetes and the systemic use of corticosteroids (10). In 2023, Mycoplasma pneumoniae infections became widely prevalent globally, with some suggesting that “immune debt” or “immunological disparity” are the main causes of this epidemic (11). A notable feature of this epidemic is the increased incidence of co-infections; studies have shown that Mycoplasma pneumoniae is prone to concurrent infections with Streptococcus pneumoniae or adenovirus (12). To date, there have been no reports of overlapping mucormycosis infections.This case report documents, for the first time, an exceedingly rare co-infection of mucormycosis and Mycoplasma pneumoniae in a child newly diagnosed with diabetes. This complex clinical scenario underscores the evolving patterns of infectious diseases in susceptible populations, challenges existing medical paradigms, and necessitates a re-evaluation of diagnostic protocols and therapeutic strategies.

This case report describes two patients who presented concurrently with symptoms of diabetes and respiratory tract infections, and were definitively diagnosed with Type 1 diabetes mellitus complicated by Mycoplasma pneumoniae. Further examination for Case 1 using an electronic bronchoscope revealed bleeding and airway obstruction, leading to a strong suspicion of mucormycosis. The gold standard for diagnosing fungal diseases is histopathological biopsy (13), but given the observed bleeding, the procedure carried high risks. Next-generation metagenomic sequencing can detect pathogenic microorganisms without bias and has been reported to detect mucormycosis in patients with acute myeloid leukemia (14). In this case, metagenomic next-generation sequencing successfully identified the pathogens, thereby highlighting the significance of modern molecular techniques in clinical diagnostics. It also underscores the importance of comprehensive pathogen detection in critically ill children with infections, enabling timely identification and management of co-infections involving multiple pathogens. Although Case 2 lacked definitive microbiological evidence, the presence of irregular cavitary lesions, subpleural nodules, luminal narrowing at the site of the lesion, and pulmonary vascular destruction and filling defects on contrast-enhanced CT provided crucial diagnostic clues for mucormycosis. Given a history of corticosteroid use, poor glycemic control, and clinical manifestations, a presumptive diagnosis of mucormycosis was made (13). In future similar cases, we recommend performing tissue biopsies whenever feasible to confirm the diagnosis, given that conditions allow.

Previous studies have found a low association between Type 1 diabetes and fungal diseases, with such cases accounting for 13% of the 157 reported cases of mucormycosis (15). In India, COVID-19-related mucormycosis infections predominantly involve the nasal, orbital-brain, and skin areas, and are more common in diabetic populations (9). Case 1 was initially diagnosed with diabetic ketoacidosis (DKA). Poor glycemic control, attributed to economic constraints and non-adherence to treatment, was noted, but the condition was eventually managed through rigorous glucose control. Case 2, although not initially diagnosed with DKA, had poor glycemic control and ultimately succumbed to the illness. Studies have shown that the environment of DKA is conducive to the growth and proliferation of mucormycosis (16). Therefore, we emphasize the importance of strict glycemic management in clinical practice to mitigate the risk of mucormycosis. In Case 1, corticosteroids were used for severe Mycoplasma pneumoniae pneumonia but were promptly discontinued upon fungal detection. In Case 2, corticosteroids were withheld considering the potential for fungal infection. We closely monitor infection risks during corticosteroid use and adjust treatment plans in a timely manner based on the patient's condition. In line with recommendations for corticosteroid use in COVID-19-related mucormycosis, we highlight the importance of glucose monitoring and judicious corticosteroid therapy (17). Both cases in this report had a history of corticosteroid use, indicating the need for vigilance regarding the possibility of mucormycosis in clinical practice and the necessity of making decisions after weighing therapeutic benefits against potential risks.

Studies have indicated that chest pain and hemoptysis may suggest aspergillosis or mucormycosis. The two patients we report on presented with fever and cough; neither experienced chest pain, and only Case 2 had massive hemoptysis before death. Chest imaging showed consolidation in the upper or middle and lower lobes of the right lung, without the specific “reversed halo sign”. There have been case reports of patients with COVID-19 who developed mucormycosis, presenting with a cavity in the lingular segment of the left upper lobe, which ultimately led to clinical deterioration and death (18). We hypothesize that Mycoplasma pneumoniae-related mucormycosis lacks specificity in clinical presentation and imaging characteristics, and that cavity formation may be associated with adverse outcomes. We hypothesize that mucormycosis associated with Mycoplasma pneumoniae could occur in newly diagnosed diabetic patients, with children potentially being a high-incidence group, mainly presenting as pulmonary mucormycosis. This hypothesis requires further research for confirmation.

Macrolide-resistant Mycoplasma pneumoniae (MRMP) infection represents a severe and widespread clinical challenge. Despite its prevalence, macrolides remain the first-line therapeutic choice. For children aged 8 years and older, tetracycline antibiotics may be considered. For those younger than 8 years, cautious use with informed consent is essential. In this case, Case 1 was treated with tetracycline antibiotics following approval from the ethics committee and consent from the pharmacy and therapeutics committee. This decision exemplifies the cautious approach required in complex clinical scenarios. In the context of Mycoplasma pneumoniae infection. Overactivation of the immune system may lead to immunosuppression, thereby creating conditions favorable for Mucorales invasion. Future research should further explore these mechanisms to develop more effective preventive and therapeutic strategies.

Current guidelines for pediatric Mycoplasma pneumoniae pneumonia and mucormycosis lack specific recommendations for concurrent infections, particularly in children. While adult guidelines advocate surgical intervention and amphotericin B lipid complex as first-line treatments for mucormycosis, pediatric guidelines are less defined. The expert consensus on pediatric invasive pulmonary fungal infections suggests a combination of amphotericin B and posaconazole for pulmonary mucormycosis but lacks guidance on surgical treatment (19). Due to drug shortages, we used amphotericin B deoxycholate instead of the preferred liposomal amphotericin B. Despite its higher side-effect profile, it effectively controlled mucormycosis progression. We also administered posaconazole for its efficacy and lower toxicity. The treatment plan was individualized based on guidelines, literature, and the patient's condition. Neither patient underwent surgery; instead, both received a comprehensive regimen including antifungal therapy, anti-Mycoplasma pneumoniae treatment, and glycemic control. This approach provides a novel strategy for managing similar cases. Case 1 showed clinical improvement, while Case 2 succumbed despite resuscitation efforts. We hypothesize that early administration of target-dose amphotericin B, inhaled amphotericin B, and posaconazole suspension, along with tetracycline antibiotics, may have contributed to Patient 1's improved prognosis. Further studies are needed to establish optimal treatment approaches for pediatric Mycoplasma pneumoniae-associated mucormycosis.

Regrettably, the family of Case 1 ceased further treatment due to financial reasons. A retrospective study evaluating the economic burden on patients with mucormycosis indicated that the duration of treatment for the disease ranged from 90 to 180 days, with hospitalizations lasting between 22 and 95 days, placing a significant financial strain on patients (20). The underlying primary condition already necessitates long-term treatment, and the concurrent mucormycosis infection further increases the financial burden on the patient. Thus, there is a clinical need for novel drugs to alleviate the economic burden on patients.

Limited clinician awareness of mucormycosis may underestimate its incidence. Disease severity and diagnostic delays contribute to high mortality rates. Amid the global outbreak of Mycoplasma pneumoniae pneumonia, awareness of fungal diseases must be heightened. The co-occurrence of these conditions can lead to significant morbidity and mortality. No guidelines exist for pediatric mucormycosis, and limited data in the literature pose challenges for clinical management. We report the first case of concurrent mucormycosis and Mycoplasma pneumoniae pneumonia, highlighting the need for evidence-based approaches to improve outcomes.

## Data Availability

The original data for this manuscript could be available on reasonable request from the corresponding author.

## Supplementary material

Supplementary Image 1 Timeline chart of Case 1.

Supplementary Image 2 Timeline chart of Case 2.
