# Supplementary figures and images for: Case Report: Co-infection of mucormycosis with mycoplasma pneumoniae in children with diabetes mellitus: report of two rare cases
Source: Front Pediatr. 2025 May 12;13:1516117. doi: 10.3389/fped.2025.1516117 (PMC12104277; doi:10.3389/fped.2025.1516117)

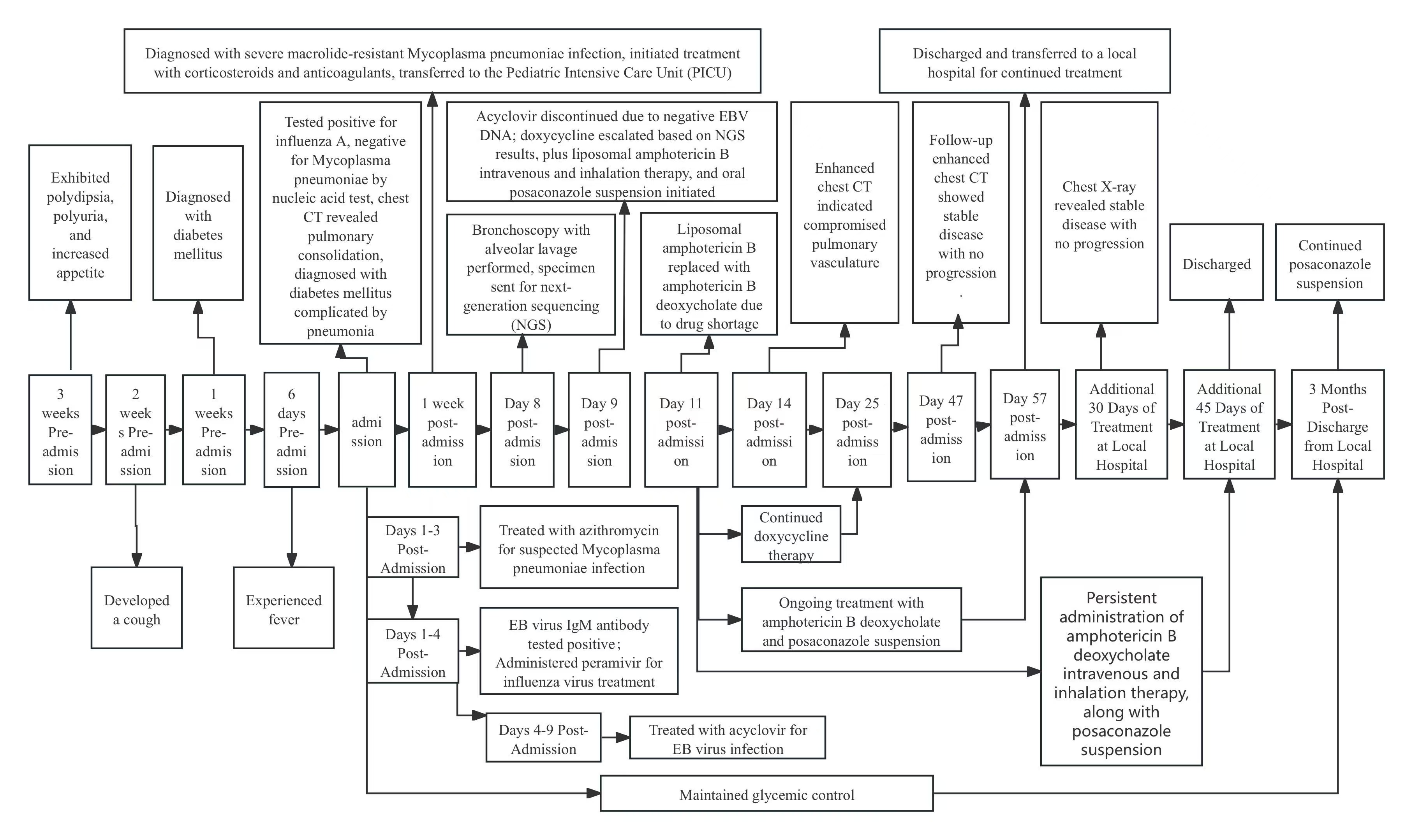

Supplement: Supplementary Image 1 — Timeline chart of Case 1. [file Image1.jpeg]

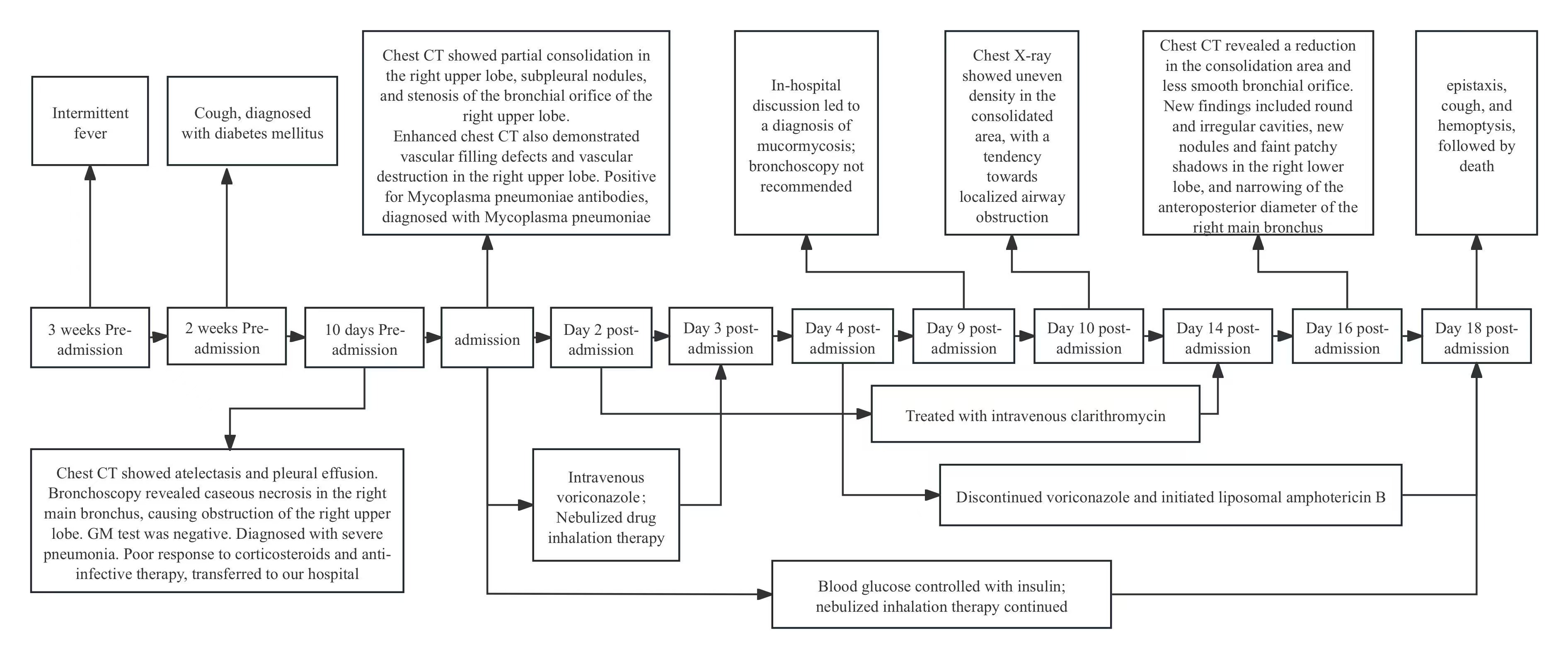

Supplement: Supplementary Image 2 — Timeline chart of Case 2. [file Image2.jpeg]
