# Supplementary material for: Case Report: Co-infection of mucormycosis with mycoplasma pneumoniae in children with diabetes mellitus: report of two rare cases
Source: Front Pediatr. 2025 May 12;13:1516117. doi: 10.3389/fped.2025.1516117 (PMC12104277; doi:10.3389/fped.2025.1516117)
Supplement: Supplementary file 3 [file Datasheet1.pdf]

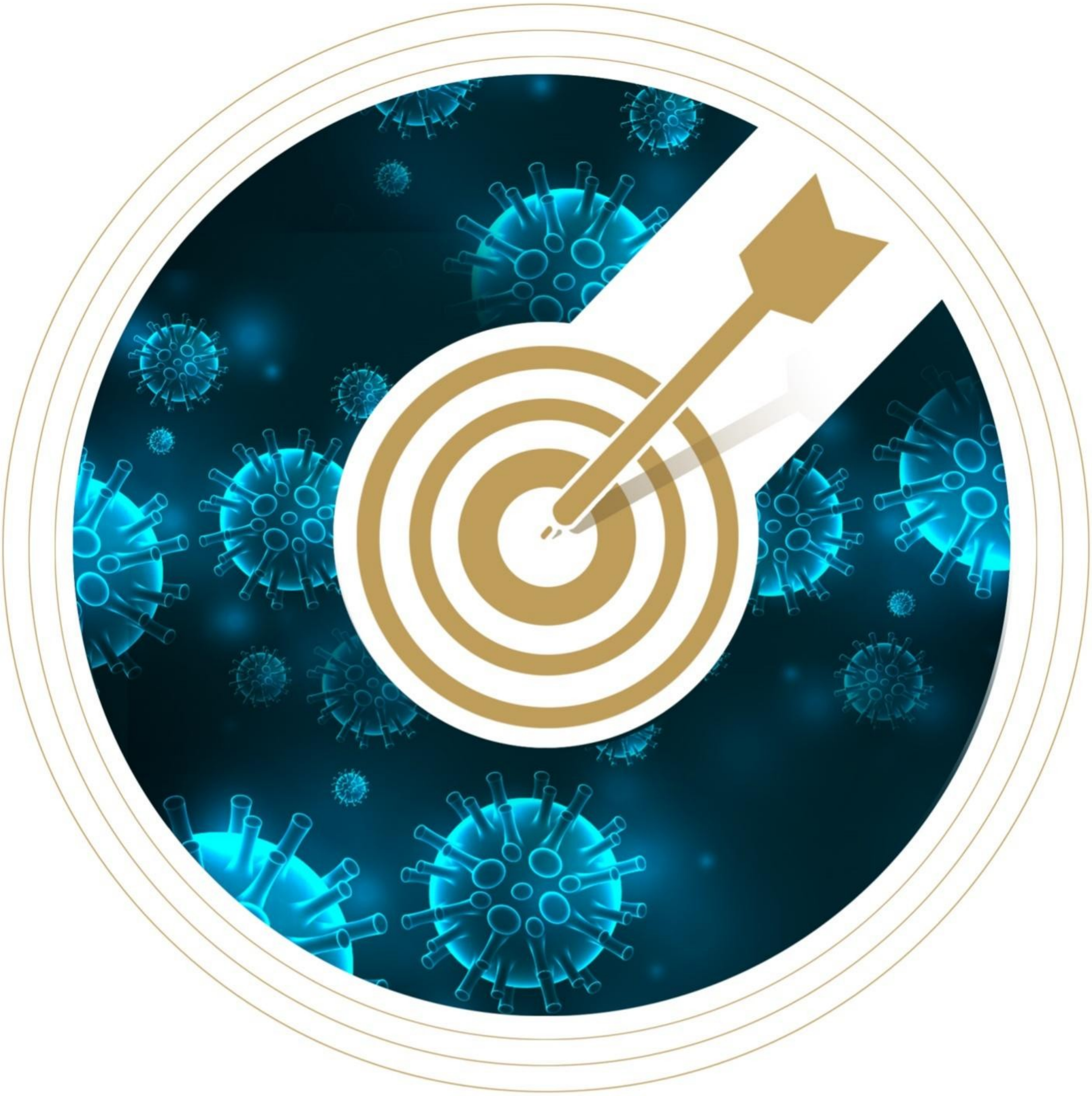

High-throughput sequencing of infectious pathogens (® PIM)

Test report: TEST REPORT

Patient name: Patient 1

Report number: 24JS01517

Date of inspection: 12-28,2023

surveillance project: Pathogenic microorganism detection-DNA detection

We only serve the infection di-
agnosis career www.genskey.com

1. Sample information

Information about the subject

** surname and personal name:	Patient 1	** sex:	Man	** age:	6 year
** telephone:	-	Admission number:	-	Bed number:	-

Sample information

Inspection hospital:	A children's hospital	Inspection physician:	-	Department ward:	Paediatrics ICU
Sampling date:	2023-12-28	Date of collection:	2023-12-28	Report date:	2023-12-29
Case No.:	24JS01517	Sample Type:	The alveolar lavage fluid		

Clinical information

White blood cell count (WBC) (109 / L): 4.69	Neutrophil ratio (%): 59.6
Lymphocyte ratio (%): 21.7	Procalcitonin (PCT) (ng/ml): -
C-reactive protein (CRP) (mg/L): 23.58	Results of the culture identification: -

Chief complaint: polydipsia, polyuria, more eating for 1 month. Cough for 2 weeks and fever for 6 days

Clinical diagnosis: diabetes mellitus, pneumonia

Clinical attention is high on the pathogen type: Mycoplasma or chlamydia, mycobacteria, parasites, fungi, viruses, and bacteria

Anti-infective drugs are timely and long: -

Sample status

Color: yellow	Character: severe turbidity with thin film	Cell count (cells / ml): 6,5,60,000
* Note: When the sample is liquefied with a clot,	Cell count results could not be obtained	

Review of test results

Bacteria: 47 Mycoplasma pneumoniae, common colonizing bacteria detected in human body (see list of suspected microecology for details)

Fungi: 15857 A. sparus, common colonization bacteria detected in human body (see suspected microecological list)

Virus: Human herpesvirus type 6B type 3

Parasites: No suspected pathogen was detected

2. Results of the DNA detection test

1. Test results of the suspected pathogens

1. List of bacteria

not detected

2. List of fungi

Category			A surname	
The species name	Sequence number	Relative abundance (%)	The species name	Sequence number
The Rhizopus genus Rhizopus	23555	66.876	*F. Rhizopus arrhizus*	15857

3. List of viruses

Category		A surname	
The species name	Sequence number	The species name	Sequence number
Rose rash virus genus Roseolovirus	4	*Human herpesvirus type 6B, Human betaherpesvirus 6B*	3

4. List of parasites

Not detected

5. List of Mycobacterium tuberculosis complex groups

Not detected

6. List of clades / chlamydia, spirochetes, ricettsii

Category			A surname	
The species name	Sequence number	Relative abundance (%)	The species name	Sequence number
Mycoplasma *Mycoplasma*	60	0.002	*Mycoplasma pneumoniae Mycoplasma pneumoniae*	47

Case No.: 24JS01517 Name: Patient 1

Pathogen explanation and sequence distribution map *

1, Mycoplasma pneumoniae (Mycoplasma pneumoniae):

The Mycoplasma family, Mycoplasma sp. This bacterium is an aerobic caustic strain without a cell wall and a common pathogen of atypical pneumonia. It can cause upper respiratory tract infection, about 5% -10% of patients will develop tracheobronchitis or pneumonia, can also lead to meningoencephalitis, arthritis and other diseases. The bacteria infection is high in late summer or autumn, children, young adults, the elderly can be infected, can be in schools or military camps outbreak epidemic.

The total number of bases in the genome of this species is 817207 (bp), and the measured total length of sequence coverage for this species is 2313 (bp), with a coverage of 0.283% and an average depth of 1.015 X.

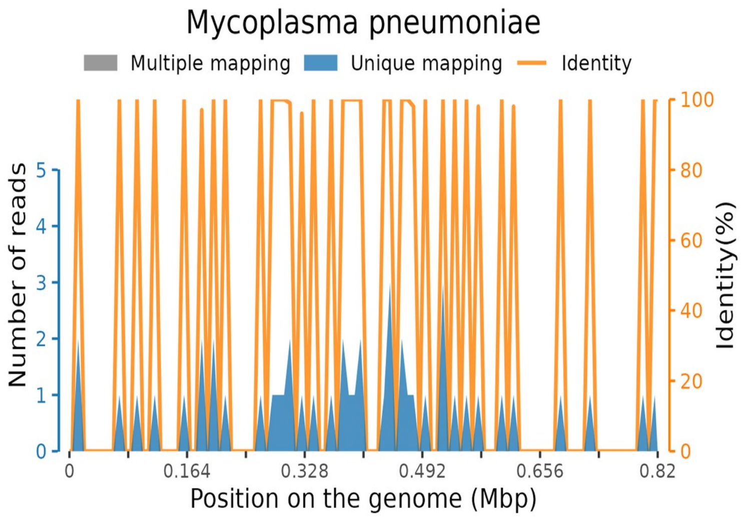

2, M. root (Rhizopus arrhizus):

Also known as Milaria, Mucoraceae, Rhizaria sp. T. oligogenum is one of the causative agents of mucormycosis. Among the hairy mold orders, A. oligogenes is currently the most common infectious agent in the western hemisphere. A. sparum is an opportunistic pathogen. Immunocompr-omised patients are most susceptible to infection, especially in patients with chronic wasting diseases such as diabetes, leukemia, long-term chemotherapy, and corticosteroid hormones. Clini-cally common is the orbital and central nervous system mucormycosis. In addition, it can also occur in the lungs, gastrointestinal tract, skin and other places. A. oligatum is responsible for 60% of mucormycosis and nearly 90% of nasal-brain infections in humans.

The total number of genome bases in this species is 40416424 (bp), and the total length of the measured sequence coverage for this species is 1065018 (bp), with a coverage of 2.6351% and an average depth of 1.054 X.

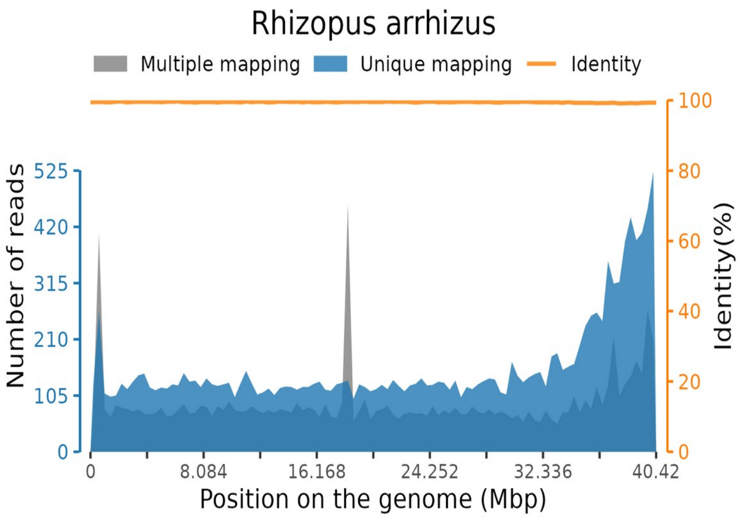

3, human herpes virus type 6B (Human betaherpesvirus 6B):

It belongs to the subfamily β herpesviruses, a member of the rose herpesvirus genus. Enveloped, double-stranded DNA viruses. Can cause infant infection and adult infection. HHV-6 infection is usually asymptomatic, and primary infection in infants and young children or immunocompromised patients can develop obvious symptoms. Severe cases, high fever (40), red maculopapular rash. Adult primary infection with HHV-6 is rare, and patients undergoing organ transplantation or immune deficiency (e. g. AIDS) can develop severe infections, such as encephalitis and interstitial pneumonia. Has a wide range of tissue sex, in human can lifelong recessive infection, is a conditional pathogen, often present pathogenic effect in immune suppression, can cause serious disease, in hematopoietic stem cell transplantation, organ transplantation and acquired immune deficiency syndrome patients can lead to the central nervous system, bone marrow, lung, digestive tract, skin and liver serious damage.

The total number of genome bases in this species is 162114 (bp), and the total length of sequence coverage for this species is 150 (bp), with a coverage of 0.0925%, and an average depth of 1.0 X.

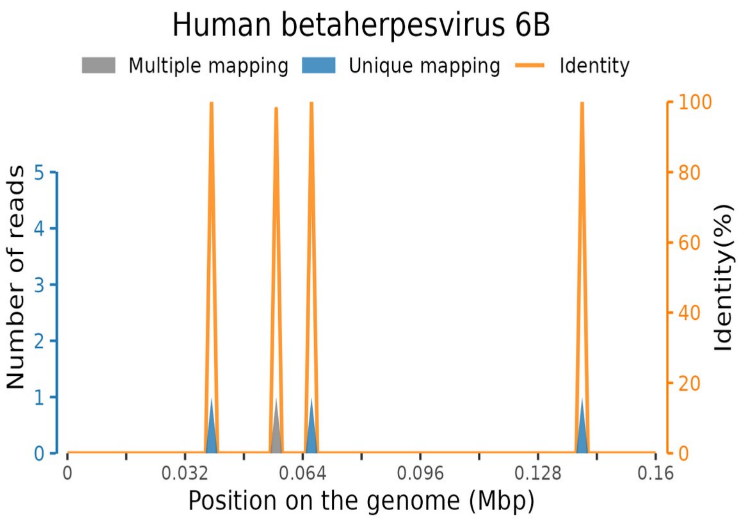

* Sequence distribution map: This section only shows the sequence distribution map of the species with three non-repeat-specific sequences detected.

2. Results of drug-resistance phenotype testing

not detected

Results of drug resistance phenotyping:

1) , "ND" means that no resistance gene is detected, "-" means that the information is unknown, and "/" means that the prediction is currently impossible to give a conclusion.
2) The column of drug resistance genes is the information of drug resistance characteristics of the corresponding antibiotic drugs, including drug resistance genes, drug resistance gene families or drug resistance mutations.
3) The drug sensitivity phenotype prediction column is the predicted drug sensitivity phenotype results obtained based on the relevant drug resistance characteristics information of the drug detected, which are divided into "resistant", "sensitive" or "/". "/" means that the drug sensitivity phenotype results cannot be predicted at present.

The above test results are for clinical reference only, and the final diagnosis should be comprehensively judged by combining other clinical test results.

3. Results of the virulence gene testing results

not detected

Results of the virulence gene test indicate that:

1) Virulence genes are related to the disease process, and the virulence genes detected in the report are for clinicians' reference only;
2) More virulence genes information is available for http://www.mgc.ac.cn/VFs/.

4. Suspected microecological test results

List of suspected microecology

Category				A surname	
Type	The species name	Sequence number	Relative abundance (%)	The species name	Sequence number
G-	Prevotella sp *Prevotella*	974123	38.922	*Oral Prevotella Prevotella oris*	310856
G+	*Streptococcus genus Streptococcus*	898224	35.89	*Oral Streptococcus Streptococcus oralis*	259579
G+	*Streptococcus genus Streptococcus*	898224	35.89	*Streptococcus infantum Streptococcus infantis*	142129
G+	Rothia *Rothia*	314940	12.584	*R. mucxae Rothia mucilaginosa*	312404
G-	Veillonella *Veillonella*	64310	2.57	*S. vircoccus Veillonella dispar*	47470
G+	Streptococcus granulosa *Granulicatella*	40416	1.615	*Adjacent to S. granulans Granulicatella adiacens*	37829

G-	*CO 2 Capnocytophaga*	27435	1.096	*Granular carbon dioxide fib - roophilia Capnocytophaga gr - anulosa*	15250
G+	- *Schaalia*	19112	0.764	*Cogenic Actinomycetes Schaa - lia odontolytica*	18173
G-	Campylobacter *Campylobacter*	2413	0.096	*Concified Campylobacter Cam - pylobacter concisus*	2346
G-	*Prevosoides genus Al - loprevotella*	617	0.025	*Prevorella ana ana Alloprev - otella tannerae*	442
fun	Candida *Candida*	11107	31.534	*And Candida albicans Candida albicans*	10322

★Compared with other microecological species, the pathogen is more prominent in the relative abundance of the genus accounting for more than 50%, and a single species accounts for more than half of the genus, which should be paid attention to. Please consider the clinical symptoms.

Explanation of the suspected microecological species

1) *Oral Prevotella species (Prevotella oris):*

Gram-negative obligate anaerobes, Prevotella spp. It is the normal human body of the oral cavity, female reproductive tract and other parts of the normal flora, but also can cause the endogenous infection of these parts. There have been reports of Prevotella species isolated in the blood of tumor patients.

2) *Oral Streptococcus (Streptococcus oralis):*

Gram-positive cocci, Streptococcus genus, belong to one of the slow streptococcus groups. Most delayed streptococcus groups are commonly colonized by oral cavity, digestive tract and female reproductive tract. This group of streptococci can instantly colonize normal skin, and such bacteria isolated from blood cultures may be contaminating bacteria. At the same time, these bacteria are also the most common pathogens found in bacterial endocarditis. In patients with granulocytosis, where immunosuppression occurs after chemotherapy, the delayed streptococcal species can often cause pathogenic sepsis and pneumonia in such patients.

3) *Streptococcus infantis (Streptococcus infantis):*

The Gram-positive cocci, Streptococcus sp. Belongs to one of the slow disease streptococcus group. Most delayed streptococcus groups are commonly colonized by oral cavity, digestive tract and female reproductive tract. This group of streptococci can instantly colonize normal skin, and such bacteria isolated from blood cultures may be contaminating bacteria. At the same time, these bacteria are also the most common pathogens found in bacterial endocarditis. In patients with granulocytosis, where immunosuppression occurs after chemotherapy, the delayed streptococcal species can often cause pathogenic sepsis and pneumonia in such patients.

4) *Rhoxi (Rothia mucilaginosa):*

Gram-positive cocci, Rhodella sp. It is a normal flora existing in the oral cavity, which can be isolated from the nasopharyngeal cavity and bronchial secretions. It is also a conditional pathogen. For isolates from blood, deep tissues, it is usually considered to be pathogenic bacteria and can cause a variety of human infections, such as endocarditis, bacteremia,

peritonitis, sepsis, etc.

5) *S. coccus (Veillonella dispar):*

Gram-negative micrococcus, and Veronella sp. It is the normal flora of the oral cavity, pharyngeal region, gastrointestinal tract and female reproductive tract. Is a conditional pathogen (not strong). Reported that it can cause artificial joint infection and endocarditis.

6) *Adjacent to let (Granulicatella adiacens):*

Gram-positive cocci, Streptococcus spp. It is a normal flora in the human pharynx, reproductive system and intestinal tract. It is an opportunistic pathogen. It can cause infective endocarditis and other diseases, and the incidence and mortality of infection can exceed the grass green streptococcus and Enterococcus. If sterile sites such as blood, deep tissue, implanted prosthetic devices, abscess pus, especially in immunocompromised patients, drug susceptibility test should be performed.

7) *Granular carbon 2 dioxide (Capnocytophaga granulosa):*

Gram-negative elongbacter, carbon 2 fibroia sp. It is the normal flora of human oral cavity, an opportunistic pathogen, related to periodontitis, which can cause sepsis and other infections in patients with normal immunity or immune deficiency (mainly granulocytopenia), such as endocarditis, endometritis, osteomyelitis, abscess, peritonitis and keratitis. Carbon CO can cause periodontitis in adults.

8) *Caries, Actinobacteria (Schaalia odontolytica):*

Also named Actinomyces odontolyticus, Gram-positive branching or club bacteria, Actinomyces sp. Actinomycetes are a major component of the dental plaque community in the healthy population and are also associated with infections such as dental caries, pulpitis, odontogenic abscesses, and dental implants. Actinomycetes and associated bacteria usually cause mixed infections. Actinomycosis can cause chronic granulomatous diseases in the head and neck, lung, abdominal and cavity and pelvic cavity, mainly caused by various actinobacteria, especially yi, gi and Gravenni, which are also reported to be associated with Propionibacterium. The bacterium was initially isolated from the teeth and saliva. Foreign cases reported that it can cause systemic severe infections such as peritonitis, brain abscess and lung disease. However, because it is difficult to accurately distinguish actinomycetes at the level of species, rare related cases were reported in China.

9) *Concise Campylobacter (Campylobacter concisus):*

Campylobacter. Mainly cause intestinal infection, can cause periodontal disease and head, neck, internal deep infection and sepsis.

10) *Prevotella tanans (Alloprevotella tannerae):*

Prevonoides spp. Obligate rod-shaped anaerobes. Isolation from plaque in the human gingival crevice can cause periodontitis. No report of infection caused by this bacterium in China.

11) *Candida albicans (Candida albicans):*

Candida, a yeast-like species, also known as Candida albicans. Candida albicans is widely distributed in nature, and can also be isolated from the normal oral cavity, skin, gastrointestinal tract, and urogenital tract. It is usually not pathogenic, and it is an important opportunistic pathogen. The use of antimicrobial drugs, immunosuppression (blood tumor, solid organ or hematopoietic stem cell transplantation, chemotherapy), tumor, diabetes and so on are the risk factors of the bacterial infection. It can lead to oral candidiasis and candida vaginitis, it is often difficult to distinguish invasive infection from asymptomatic colonization, and the clinical manifestations can be from local mucosal lesions (local excessive proliferation and invasive infection) to disseminated infection (blood-borne infection).

explanation of nouns:

® Number of sequences: the number of sequences matching to the pathogen is related to the load load

of the pathogen itself, the amount of nucleic acid extraction, and the proportion of human sequences. A higher number of sequences indicates the higher confidence of the pathogen detected in the specimen.

® Relative abundance: the proportion of the microorganism in the same type of microorganisms detected in the whole specimen, the higher the abundance, the higher the proportion in the same type of microorganisms.

® Coverage: indicates the ratio of the detected nucleic acid sequence of the microorganism covering the entire gene sequence of the microorganism, and the high coverage indicates the high ratio detected by the whole genome of the microorganism.

® Multiple Mapping: Multiple alignment, which can compare reads to two or more species.

® Unique Mapping: only alignment, only alignment of reads to a species.

5. Description of test results

This method, just like other detection methods, has its own detection capability and detection range. The microorganisms not reported in the test results do not mean that they must not exist in the sample. The reasons include but are not limited to:

1) The proportion of human nucleic acid in the sample is too high, and the load of the tested pathogen in the sample is lower than the product detection limit;
2) For new pathogens or rare pathogens, the database may not be included;
3) The use of anti-infective drugs before sampling will lead to a decrease in the microbial content, which may affect the detection of pathogens;
4) Sample transportation conditions are not appropriate, resulting in nucleic acid degradation and sample loss;
5) Infection caused by the RNA virus, the DNA process cannot be effectively detected, please pay attention to the RNA process test results;

There are many causes of clinical fever (refer to the following table below), among which infectious fever accounts for about 40% and non-infectious fever accounts for about 60%. Clinicians are requested to make comprehensive judgment based on other clinical diagnoses and symptoms.

Fever nature	Cause of disease	Disease
Infectious fever (~40%)	Various pathogens (bacteria, viruses, mycoplasma, chlamydia, spirochetes, rickettsia, and parasites, etc.)	Acute and chronic systemic or focal infections
Non-infectious fever (~60%)	Blood disease	Lymphoma, malignant histiocytosis, hemophagocytic syndrome, acute myeloid leukemia, multiple myeloma, etc
	Allergy reaction and connective tissue disease	Rheumatic fever, drug fever, systemic lupus erythematosus, dermatomyositis, Behcucet's disease, ankylosing spondylitis, autoimmune hepatitis, reactive arthritis, adult Still disease, etc
	Solid tumor	Liver and central nervous system metastases, renal cell cancer, liver cancer, colon cancer, pancreatic cancer, etc

3. Cell count results

Metric	Sample data	Host background distribution of the same-type samples
Human cell concentration (cells / ml)	6560000	83.66%

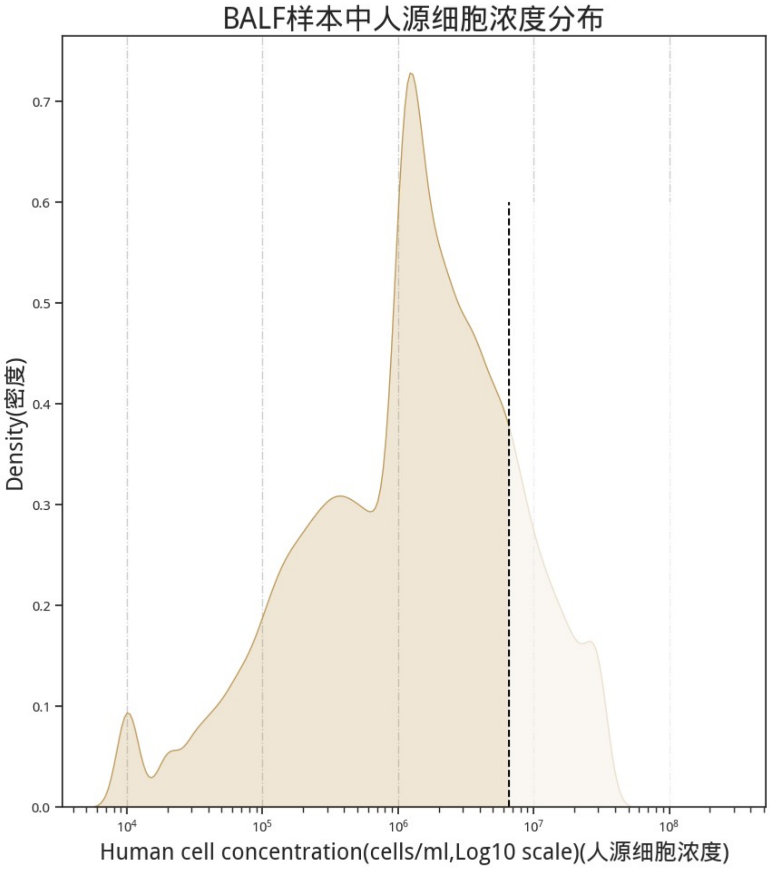

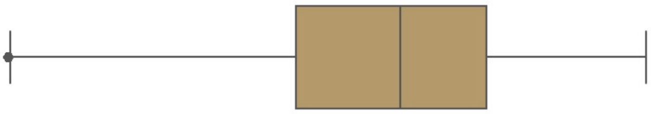

Case No.: 24JS01517 Name: Patient 1

4. Sequencing quality

	Quality control parameters	Sample data
Experimental quality control	Internal reference	Qualified
	Nucleic acid extraction concentration (ng / μ L)	0.262
	Library Concentration (ng / μ L)	60.4
	Yin control	Qualified
	Whether to go to the host	Yes
Data quality control	Total amount of data	35191821
	Number of non-humanized sequences	4905622
	Q30(%)	92.8564

Quality Distribution of Base

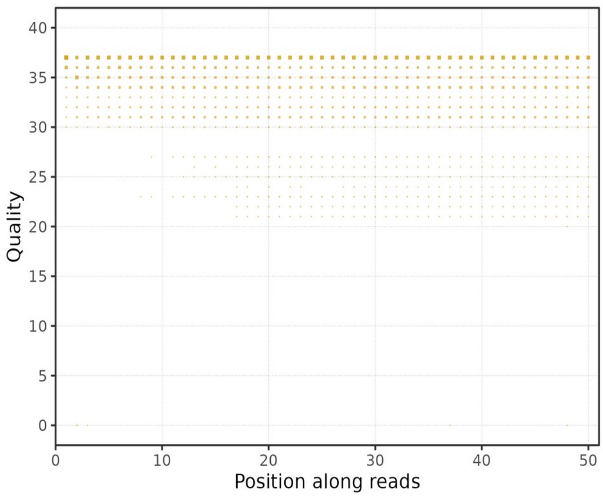

Test person: 俏元元 auditor: 刘艳 Report date: 12-29,2023

天津金匙医学检验实验室 报告专用章

disclaimer:

1) The results of this report are only responsible for the samples submitted for inspection at this time. If you have any doubt, please contact us within 7 working days after receiving the report;

2) The test results are available for clinical reference only, and the final clinical diagnosis and treatment plan need to be combined with the comprehensive clinical information of the subject person The doctor came to;

3) Because the subject is aware of the possible mental pressure and psychological burden of the result, the testing institution shall not bear joint and several liability;

4) The test shall keep the results confidential and protect the privacy of the subject according to law, but the testing institution shall not bear the corresponding responsibility for the information leakage occurring due to the personal reasons of the subject.

Electronic report query method:

Way one:

Wechat scan code report QR code, input the name of the subject and the reserved mobile phone number, obtain the SMS verification code, you can query;

Way two:

Search for "Golden Medical Laboratory", click "Pathogen Detection-Metagenomic Report Query", enter the name of the subject and the reserved mobile phone number, obtain the SMS verification code, you can query.

5. Methodological introduction

The high-throughput detection product of ® infection was tested based on nucleic acids in the sample to identify the suspected pathogenic microorganisms in the sample. The detectable range includes 12895 bacteria, 11120 viruses, 1582 fungi, 312 parasites, and 177 common pathogenic bacteria and 184 mycoplasma / chlamydia in the mycobacterium complex group. Meanwhile, detect the suspected resistance genes present in the identified samples, thus predicting the possible resistance of the bacteria in the samples based on the resistance genes. This test report provides all microorganisms with valid data detected in the sample and assists the clinician by interpreting the report. It is suitable for patients with unexplained fever, difficulty and immune deficiency.

We used high-throughput sequencing technology to analyze the microbial nucleic acid sequences in the samples and identify the microorganisms by comparing them with the nucleic acid sequences of the existing microorganisms in the database. Using machine learning (machine learning), synchronous error exploration (error modeling), background clearing (denoising) and exact sequence inference (exact sequence inference), and strict quality control system automatically eliminate false positive results.

techniqueflow

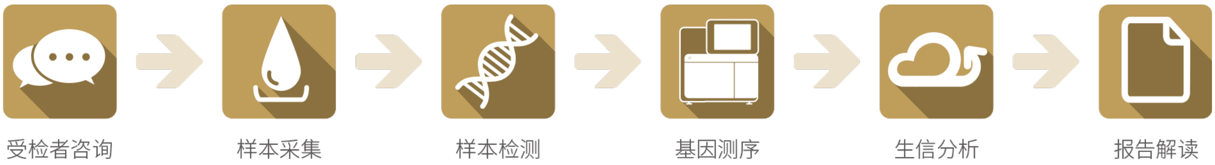

Distribution of human microorganisms

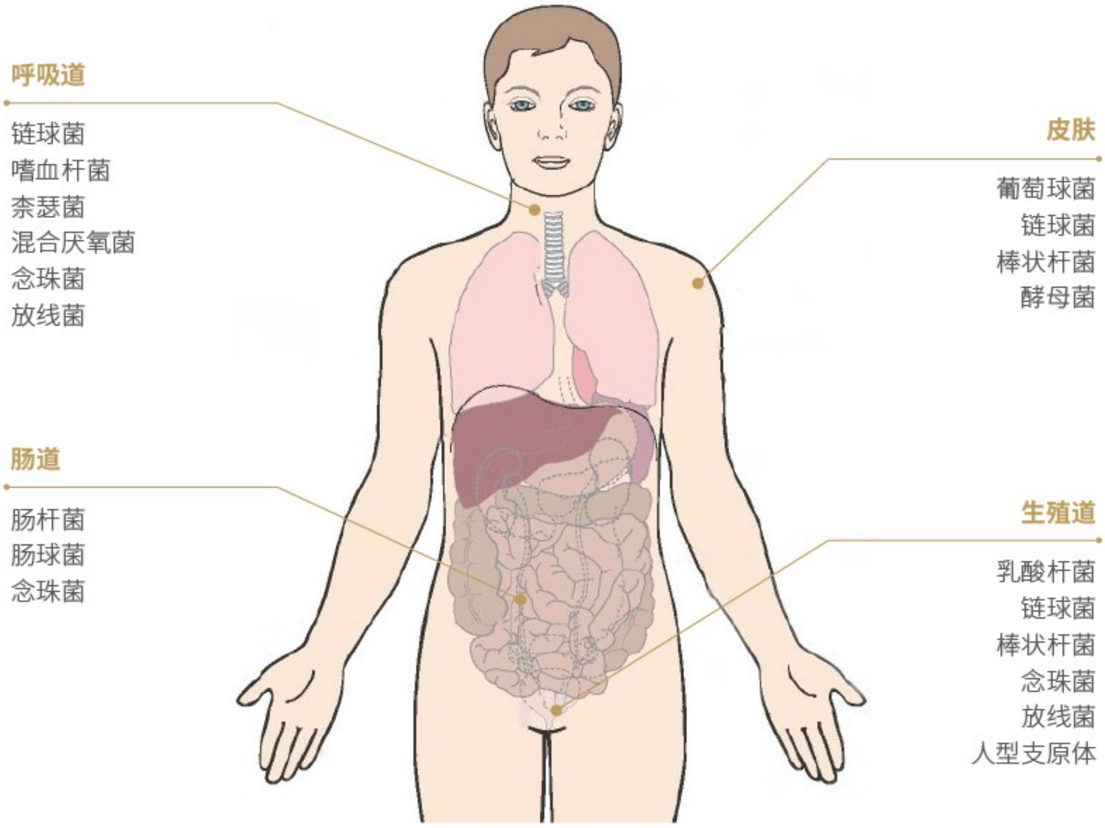

Case No.: 24JS01517 Name: Patient 1

Six, microbial antibacterial spectrum

细菌	青霉素G	苯唑西林	氨苄西林	阿莫西林	阿莫西林-克拉维酸	氨苄西林-舒巴坦	哌拉西林-他唑巴坦	厄他培南	亚胺培南-西司他丁	美罗培南	氨曲南	环丙沙星	左氧氟沙星	莫西沙星	头孢唑啉	头孢替坦	头孢西丁	头孢呋辛	头孢噻肟	头孢曲松	头孢他啶	头孢吡肟	头孢他啶-阿维巴坦	头孢氨苄	庆大霉素	妥布霉素	阿米卡星	克林霉素	红霉素	阿奇霉素	多西环素	米诺环素	替加环素	万古霉素	替考拉宁	利奈唑胺	多黏菌素B	TMP-SMX	磷霉素（静脉）	磷霉素（口服）	甲硝唑
粪肠球菌(敏感)	++	0	++	++	+	+	+	0	+	±	0	±	+	+	0	0	0	0	0	±	0	0	0	0	±	0	0	0	0	0	±	±	+	++	++	+	0	0	±	±	0
屎肠球菌(敏感)	±	0	±	±	±	±	±	0	0	0	0	0	0	±	0	0	0	0	0	0	0	0	0	0	±	0	0	0	0	0	±	±	+	±	±	++	0	0	±	±	0
粪肠球菌(VRE)	±	0	±	±	±	±	±	0	0	0	0	0	0	0	0	0	0	0	0	0	0	0	0	0	±	0	0	0	0	0	±	±	±	0	0	++	0	0	±	±	0
屎肠球菌(VRE)	±	0	±	±	±	±	±	0	0	0	0	0	0	0	0	0	0	0	0	0	0	0	0	0	±	0	0	0	0	0	±	±	±	0	0	++	0	0	±	±	0
MSSA	±	++	±	±	+	+	+	+	+	+	0	0	+	+	++	+	+	+	+	+	0	+	0	+	±	0	0	+	±	±	+	+	+	+	+	+	0	+	±	0	0
MRSA	0	0	0	0	0	0	0	0	0	0	0	0	0	0	0	0	0	0	0	0	0	0	0	0	±	0	0	±	0	0	+	+	+	++	++	++	0	+	±	0	0
路邓葡萄球菌	±	++	±	±	+	+	+	+	+	+	0	0	+	+	++	+	+	+	+	+	0	+	0	+	±	0	0	+	+	+	+	+	+	+	+	+	0	+	0	0	0
咽峡炎链球菌	++	+	+	+	+	+	+	+	+	+	0	±	±	±	+	+	+	+	+	+	±	+	±	+	±	0	0	+	±	±	±	±	+	+	+	+	0	0	0	0	0
化脓性链球菌（A）	++	+	+	+	+	+	+	+	+	+	0	±	±	±	+	+	+	+	+	+	+	+	+	+	±	0	0	+	±	±	0	0	+	+	+	+	0	±	0	0	0
无乳链球菌(B)	++	+	+	+	+	+	+	+	+	+	0	±	±	±	+	+	+	+	+	+	+	+	+	+	±	0	0	+	±	±	0	0	+	+	+	+	0	±	0	0	0
肺炎链球菌	++	+	+	+	+	+	+	+	+	+	0	±	+	+	+	+	+	+	+	+	+	+	+	+	0	0	0	+	±	±	±	±	+	+	+	+	0	±	0	0	0
产单核李斯特菌	+	0	++	+	+	+	+	0	0	±	0	0	+	+	0	0	0	0	0	0	0	0	0	0	±	0	0	0	0	0	0	0	0	0	0	+	0	+	0	0	0
诺卡菌	0	0	0	0	±	0	0	0	++	±	0	0	0	0	0	0	0	0	±	±	0	0	0	0	0	0	++	0	0	0	0	±	0	0	0	+	0	++	0	0	0
气单胞菌属	0	0	0	0	±	±	+	+	+	+	+	+	+	+	0	0	0	0	+	+	+	+	+	0	+	+	+	0	0	0	+	+	+	0	0	0	0	+	0	0	0
阴沟肠杆菌	0	0	0	0	0	0	0	+	+	+	0	+	+	+	0	0	0	0	0	0	0	+	+	0	+	+	+	0	0	0	0	0	+	0	0	0	0	+	±	±	0
大肠埃希菌(敏感)	0	0	±	±	+	+	++	+	+	+	+	+	+	+	+	+	+	+	+	++	+	+	+	±	+	+	+	0	0	±	±	±	+	0	0	0	0	±	+	+	0
大肠埃希菌ESBL	0	0	0	0	0	0	0	++	++	++	0	±	±	±	0	+	+	0	0	0	0	0	+	0	±	±	±	0	0	0	0	0	+	0	0	0	0	±	+	+	0
大肠埃希菌/克雷伯菌KPC	0	0	0	0	0	0	0	0	0	0	0	0	0	0	0	0	0	0	0	0	0	0	++	0	±	±	±	0	0	0	0	0	+	0	0	0	±	±	±	±	0
大肠埃希菌/克雷伯菌MBL	0	0	0	0	0	0	0	0	0	0	±	±	±	0	0	0	0	0	0	0	0	0	0	0	±	±	±	0	0	0	0	0	+	0	0	0	±	0	±	±	0
肺炎克雷伯菌	0	0	0	0	+	+	++	+	+	+	+	+	+	+	+	+	+	+	+	++	+	+	+	±	+	+	+	0	0	0	±	±	+	0	0	0	0	±	+	+	0
克雷伯属	0	0	0	0	0	0	0	+	++	++	0	±	±	±	0	±	±	0	0	0	0	0	+	0	±	±	±	0	0	0	0	0	+	0	0	0	0	±	+	+	0
摩根菌属	0	0	0	0	0	0	++	+	±	+	+	+	+	0	0	+	+	0	+	++	+	+	+	0	+	+	+	0	0	0	0	0	0	0	0	0	0	±	0	0	0
奇异变形杆菌	0	0	+	+	+	+	+	+	±	+	+	+	+	+	+	+	+	+	+	+	+	+	+	±	+	+	+	0	0	0	0	0	0	0	0	0	0	±	+	+	0
普罗威登菌属	0	0	0	0	+	+	+	++	±	++	+	+	+	+	0	+	+	0	+	+	+	+	+	0	±	±	+	0	0	0	0	0	0	0	0	0	0	±	+	+	0
沙门菌属	0	0	±	±	0	0	0	+	+	+	0	±	±	±	0	0	0	0	+	+	+	+	+	0	0	0	0	0	0	+	0	0	0	0	0	0	0	±	0	0	0
沙雷菌属	0	0	0	0	0	0	+	+	+	+	+	+	+	+	0	+	0	0	+	+	+	+	+	0	+	+	+	0	0	0	0	0	+	0	0	0	0	+	±	±	0
巴尔通体属	0	0	0	0	0	0	0	0	0	0	0	0	0	0	0	0	0	0	0	0	0	0	0	0	++	0	0	0	+	++	++	+	0	0	0	0	0	0	0	0	0
百日咳鲍特菌	0	0	0	0	0	0	0	0	0	0	0	0	0	0	0	0	0	0	0	0	0	0	0	0	0	0	0	0	+	++	0	0	0	0	0	0	0	+	0	0	0
伯氏疏螺旋体	+	0	++	++	+	+	0	0	0	0	0	0	0	0	0	0	0	+	+	++	0	0	0	0	0	0	0	0	+	+	++	+	0	0	0	0	0	0	0	0	0
布鲁菌属	0	0	0	0	0	0	0	0	0	0	0	+	0	0	0	0	0	0	0	0	0	0	0	0	+	0	0	0	0	0	+	+	0	0	0	0	0	+	0	0	0
贝纳立克次体	0	0	0	0	0	0	0	0	0	0	0	+	+	+	0	0	0	0	0	0	0	0	0	0	0	0	0	0	+	+	++	+	0	0	0	0	0	+	0	0	0
流感嗜血杆菌	0	0	±	±	++	++	+	+	+	+	+	+	++	++	0	+	+	+	++	++	+	+	+	0	0	0	0	0	0	+	+	+	+	0	0	0	0	+	+	0	0
军团菌属	0	0	0	0	0	0	0	0	0	0	0	++	++	++	0	0	0	0	0	0	0	0	0	0	0	0	0	0	++	++	+	+	+	0	0	0	0	+	0	0	0
钩端螺旋体	++	0	+	+	+	+	+	+	+	+	+	+	+	+	0	0	0	0	++	++	0	+	+	0	0	0	0	0	+	++	++	+	0	0	0	0	0	0	0	0	0
卡他莫拉菌	0	0	0	0	++	+	+	+	+	+	+	+	+	+	0	+	±	+	+	+	+	+	+	0	0	0	0	0	+	+	+	+	0	0	0	0	0	+	0	0	0
脑膜炎奈瑟菌	+	0	++	++	0	0	+	+	+	+	+	0	0	0	0	0	0	0	+	++	+	+	+	0	0	0	0	0	0	0	0	0	0	0	0	0	0	0	0	0	0
创伤弧菌	0	0	0	0	0	0	0	0	0	0	0	+	+	0	0	0	0	0	+	+	+	+	+	0	0	0	0	0	0	0	+	+	0	0	0	0	0	0	0	0	0
鲍曼不动杆菌	0	0	0	0	0	±	±	0	±	±	0	±	±	±	0	0	0	0	0	0	±	±	±	0	0	0	±	0	0	0	0	±	0	0	0	0	±	±	0	0	0
洋葱伯克霍尔德菌	0	0	0	0	0	0	0	0	±	±	0	0	±	0	0	0	0	0	0	0	±	±	+	0	0	0	0	0	0	0	0	±	0	0	0	0	0	+	0	0	0
铜绿假单胞菌	0	0	0	0	0	0	++	0	+	++	+	+	+	0	0	0	0	0	0	0	++	++	+	0	+	+	+	0	0	0	0	0	0	0	0	0	±	0	±	±	0
嗜麦芽窄食单胞菌	0	0	0	0	0	0	0	0	0	0	0	0	±	±	0	0	0	0	0	0	±	0	±	0	0	0	0	0	0	0	+	+	+	0	0	0	±	++	0	0	0
衣原体属	0	0	0	0	0	0	0	0	0	0	0	+	+	+	0	0	0	0	0	0	0	0	0	0	0	0	0	0	+	+	++	+	+	0	0	0	0	0	0	0	0
肺炎支原体	0	0	0	0	0	0	0	0	0	0	0	+	++	++	0	0	0	0	0	0	0	0	0	0	0	0	0	0	±	±	++	++	+	0	0	0	0	0	0	0	0
脆弱拟杆菌	0	0	0	0	++	+	++	++	++	++	0	0	0	±	0	±	±	0	0	0	0	0	0	0	0	0	0	0	0	0	±	±	+	0	0	0	0	0	0	0	++
放线菌属	++	0	++	++	+	+	+	+	+	+	0	0	0	0	0	0	+	0	+	+	0	0	0	0	0	0	0	++	++	+	+	+	0	0	0	+	0	0	0	0	0
梭菌属	++	0	+	+	+	+	+	+	+	+	0	0	0	0	0	+	+	0	+	+	0	0	0	0	0	0	0	+	+	+	+	+	+	+	+	+	0	0	0	0	+

Case No.: 24JS01517 Name: Patient 1

真菌-1	氟康唑	伊曲康唑	伏立康唑	泊沙康唑	艾沙康唑	阿尼芬净	卡泊芬净	米卡芬净	两性霉素B
烟曲霉	0	±	++	+	++	±	±	±	+
土曲霉	0	±	++	+	++	±	±	±	0
黄曲霉	0	±	++	+	++	±	±	±	+
白念珠菌	++	+	+	+	+	++	++	++	+
耳念珠菌	0	±	±	±	±	+	+	+	±
都柏林念珠菌	++	+	+	+	+	++	++	++	++
光滑念珠菌	±	±	±	±	±	++	++	++	++
季也蒙念珠菌	++	++	++	++	+	++	++	++	++
克柔念珠菌	0	0	+	+	+	++	++	++	++
葡萄牙念珠菌	++	+	+	+	+	++	++	++	0
近平滑念珠菌	++	+	+	+	+	+	+	+	++
热带念珠菌	++	+	+	+	+	++	++	++	++

真菌-2	氟康唑	伊曲康唑	伏立康唑	泊沙康唑	艾沙康唑	阿尼芬净	卡泊芬净	米卡芬净	两性霉素B
隐球菌	++	+	+	+	+	0	0	0	++
暗色真菌	0	++	++	+	+	±	±	±	+
镰刀霉菌	0	±	±	±	±	0	0	0	±
马尔尼菲篮状菌	0	++	++	0	0	0	0	0	++
毛霉菌	0	0	0	+	+	0	0	0	++
尖端赛多孢菌	0	0	+	±	±	0	0	0	0
多育赛多孢菌	0	0	0	0	0	0	0	0	0
毛孢子菌	±	+	+	+	+	0	0	0	+
芽生菌	±	++	+	+	+	0	0	0	++
球孢子菌	++	++	+	+	+	0	0	0	++
组织胞浆菌	±	++	+	+	+	0	0	0	++
孢子丝菌	±	++	+	+	+	0	0	0	++

++	推荐,该药为一线推荐治疗药物，体外药物试验敏感，临床有效，指南推荐，《桑福德抗微生物治疗指南》推荐一线用药或可接受的替代用药
+	有活性,该药是备选药物(体外药物试验敏感，与已知有效药物或治疗上可替换药物敏感性相当，因此临床治疗可能有效。但因其过于广泛的抗菌谱、药物毒性、缺乏临床经验或缺乏治疗有效直接证据被列为二线用药。)
±	不确定,抗菌活性不确定，在某些病例，某些类型感染时有效，但在其他类型疗效不确定，或需与其他药物联合治疗，和/或因耐药而导致疗效差，且证实与治疗失败有关
0	不推荐,药物不作为其他药物的替代方案被推荐，因为可能已经存在或发生耐药、药物在感染部位渗透性差、毒副作用大或缺少临床治疗有效的数据
出处:热病-桑福德抗微生物治疗指南.2024	

病毒	腺病毒	BK病毒	SARS COV-2	巨细胞病毒	乙肝病毒	单纯疱疹病毒	甲型流感病毒	乙型流感病毒	JC病毒/PML	呼吸道合胞病毒	天花	猴痘	带状疱疹病毒
冠状病毒													
瑞德西韦	NA	NA	++	NA	NA	NA	NA	NA	NA	NA	NA	NA	NA
乙型肝炎													
恩替卡韦	NA	NA	NA	NA	++	NA	NA	NA	NA	NA	NA	NA	NA
替诺福韦(TDF和TAF)	NA	NA	NA	NA	++	±	NA	NA	NA	NA	NA	NA	NA
流感													
金刚烷胺	NA	NA	NA	NA	NA	NA	0	0	NA	NA	NA	NA	NA
巴洛沙韦	NA	NA	NA	NA	NA	NA	+	+	NA	NA	NA	NA	NA
奥司他韦	NA	NA	NA	NA	NA	NA	++	++	NA	NA	NA	NA	NA
帕拉米韦	NA	NA	NA	NA	NA	NA	+	+	NA	NA	NA	NA	NA
金刚乙胺	NA	NA	NA	NA	NA	NA	0	0	NA	NA	NA	NA	NA
扎那米韦	NA	NA	NA	NA	NA	NA	++	++	NA	NA	NA	NA	NA
疱疹病毒，CMV,VZV等													
阿昔洛韦	NA	NA	NA	0	NA	++	NA	NA	NA	NA	NA	NA	+
西多福韦	+	+	NA	++	NA	+	NA	NA	+	NA	+	+	+
泛昔洛韦	NA	NA	NA	0	NA	++	NA	NA	NA	NA	NA	NA	+
膦甲酸钠	NA	NA	NA	++	NA	+	NA	NA	NA	NA	NA	NA	+
更昔洛韦	±	NA	NA	++	NA	+	NA	NA	NA	NA	NA	NA	+
乐特莫韦	NA	NA	NA	++	NA	NA	NA	NA	NA	NA	NA	NA	NA
伐昔洛韦	NA	NA	NA	0	NA	++	NA	NA	NA	NA	NA	NA	++
缬更昔洛韦	±	NA	NA	++	NA	+	NA	NA	NA	NA	NA	NA	+
痘病毒													
特考韦瑞	NA	NA	NA	NA	NA	NA	NA	NA	NA	NA	+	+	NA

The above data are based on treatment guidelines and recommendations, in vitro susceptibility results, major sensitivity and resistance mechanisms, and exact clinical efficacy, and are recommended as routine guidance for antimicrobial therapy. Methods of bacterial resistance vary by region and by the site of infection (e. g., community acquired infection, ICU acquired infection). These differences should be taken into account when applying the table above, and the activities of some drugs may be very different from those listed in the table.

VII. References

1. Editorial Committee of Chinese Journal of Infectious Diseases. Expert Consensus on Diagnosis and Treatment of Fever [J], Shanghai Medicine, 2018. (41): 385-400.

2. Miao Qing, Yao Yumeng, Pan Jue, et al. Value of macro-gene second-generation sequencing technology for pathogen diagnosis of nontubercular Mycobacterium infection [J]. Clinical Medicine of China, 2020,27 (4): 559-562.

3. Consensus expert group on clinical application of metagenomics sequencing technology in moderate and severe infection, Sepsis and Shock Committee of Chinese Society of Research Hospitals, Microbiological toxin Committee of Chinese Society of Microbiology, et al. Expert consensus on clinical application of metagenomics sequencing technology in moderate and severe infection (first edition)
[J]. Zhonghua Critical Care Emergency Medicine, 2020,32 (05): 531-536.

4. Editorial Committee of Chinese Journal of Infectious Diseases. Expert consensus on the clinical application of second-generation sequencing technology in Chinese metagenomics to detect infecti-ous pathogens [J]. Chinese Journal of Infectious Diseases, 2020,38 (11): 681-689.

5. Handbook of Clinical Microbiology: 12th edition / (USA) Karen C. Carol (Karen C.Carroll)

6. Infectious Diseases and cerebrospinal fluid Cytology Group of Chinese Medical Association. Expert Consensus on the application of the cerebrospinal fluid metagenomics of central nervous system [J]. Chinese Journal of Neurology, 2021,54 (12): 1234-1240.

7. Branch of Bacterial Infection and Resistance Prevention of Chinese Medical Association. Expert Consensus on clinical Application and Results Interpretation of Metagenomic sequencing Technology in Respiratory Infection [J]. Chinese Journal of Clinical Infectious Diseases, 2022,15 (2): 90 -102.

8. Respiratory Society of Chinese Medical Association. Expert consensus on clinical interpretation path of Lower Respiratory tract Infection [J]. Chinese Journal of Tuberculosis and Respiratory, 2023,46 (4): 322-335.

9. Fever: Sanford antimicrobial Therapy: 53 / (US) Dai Wei Gilbert (David N.Gilbert,M.D.); Fan Hongwei, Beijing: China Union Medical College University Press, 2024.1.

10. Blauwkamp TA, Thair S, Rosen MJ, Blair L, Lindner MS, Vilfan ID, et al. Analytical and clinical validation of a microbial cell-free DNA sequencing test for infectious disease. Nat Microbiol 2019;4(4):663-674.

11. Manish Boolchandani, Alaric W. D' Souza Gautam Dantas. Sequencing-based methods and resources to study antimicrobial resistance. Nature Reviews Genetics 2019; 20:356–370.

12. ShiCL, HanP, TangPJ, et al. Clinical metagenomic sequencing for diagnosis of pulmonary tuberculosis[J]. J Infection, 2020, 81(4): 567-574.

13. LiN, CaiQ, MiaoQ, et al. High-throughput metagenomics for identification of pathogens in the clinical settings[J]. Small Methods，2021，5(1):2000792.

交付时间**更快速**　检测结果**更准确**　售后服务**更完善**

GensKey
金匙医学

北京：丰台区右安门外东滨河路甲2号第三街区产业园3号楼1层110
天津：武清开发区创业总部基地B21栋
上海：嘉定区惠平路801号2号楼101
广州：黄埔区开源大道11号科技企业加速器C5栋202-203
电话：400-0618-001
官网：www.genskey.com
邮箱：genskey@genskey.com
